# Pronuclear score improves prediction of embryo implantation success in ICSI cycles

**DOI:** 10.1186/s12884-021-03820-7

**Published:** 2021-05-05

**Authors:** Sara Stigliani, Claudia Massarotti, Francesca Bovis, Ida Casciano, Fausta Sozzi, Valentino Remorgida, Angelo Cagnacci, Paola Anserini, Paola Scaruffi

**Affiliations:** 1 UOS Physiopathology of Human Reproduction, IRCCS Ospedale Policlinico San Martino, Largo R. Benzi, 10, 16132 Genova, Italy; 2 Department of Neurosciences, Rehabilitation, Ophthalmology, Genetics, Maternal and Child Health (DINOGMI), Academic Unit of Obstetrics and Gynecology, University of Genova, Genova, Italy; 3 Department of Health Sciences (DISSAL), University of Genova, Genova, Italy

**Keywords:** Pronuclear morphology, Zygote, Blastocyst development, Implantation, Preimplantation embryo

## Abstract

**Background:**

In assisted reproduction technology embryo competence is routinely evaluated on morphological criteria but efficacy remains relatively low. Additional information could be obtained by evaluating pronuclear (PN) morphology. Up to now controversial results have been reported about the prognostic value of PN score. One of the main limitations of literature data is the use of different PN classification methods. In this regard, in 2011 the ESHRE and Alpha Scientists in Reproductive Medicine defined three PN categories to standardize zygote assessment. In this study we evaluated whether the consensus ESHRE-Alpha system for the pronuclear scoring could be an useful additional criterion to improve prediction of embryo implantation potential.

**Methods:**

This is a retrospective, longitudinal, observational, cohort study. We included 3004 zygotes from 555 women who underwent ICSI treatment at our Center between January 2014 and June 2019. The PN were categorized as score 1: symmetrical, 2: non-symmetrical, 3: abnormal. A subset of 110 zygotes did not cleaved. On day 2–3 1163 embryos were transferred, 232 arrested, and 9 were cryopreserved. Among the 1490 embryos cultured up to day 5–7, 516 became blastocysts: 123 were transferred on day 5 and 393 were cryopreserved.

Comparisons of age, cleavage and blastocyst rate, quality of embryos, implantation success among PN score groups were evaluated by chi-square test or Kruskal-Wallis test as appropriate. Potential predictors of embryo implantation were first tested in univariable analysis using generalized estimating equations taking into account correlation between embryos originated from the same patient. Then, variables potentially associated with implantation success (*P*<0.05) were included in a multivariable analysis for calculating the adjusted odds ratio (OR) and 95% confidence interval (CI).

**Results:**

There was no significant difference in patients’age, cleavage and blastulation rates, and embryo morphology among the three PNscore groups. The PN score 1-embryos had a greater implantation success respect to score 2-3-ones (OR 1.83; 95% CI 1.34-2.50, *P*=0.0001). Consistently, the pronuclear score remained predictive of implantation in top quality embryos (OR 1.68; 95%CI 1.17-2.42, *P*= 0.005).

**Conclusions:**

The consensus pronuclear score may be routinely included among criteria for embryo evaluation to increase patients’ chance of becoming pregnant.

**Supplementary Information:**

The online version contains supplementary material available at 10.1186/s12884-021-03820-7.

## Introduction

Identification of the embryo(s) with the highest implantation potential is a challenge not yet achieved in reproductive medicine, and it is a fundamental step for single embryo transfer approach. During the years, several approaches have been proposed for embryo viability evaluation, i.e. embryo morphokinetics, study of metabolic activity, prolonged culture, and both invasive and non-invasive preimplantation genetic testing [1–4]. Various embryological parameters are to some extent predictive of implantation potential. However, the overall success of these markers is still limited, with over 50 % of transferred embryos failing to implant. Even invasive preimplantation genetic testing failed to improve overall pregnancy outcomes in a randomized controlled trial [5]. Thus, the search for new and reproducible markers of embryo viability is still in progress. For instance, it has been proposed that additional information on embryo viability potential could be obtained by evaluating pronuclear (PN) morphology based on zygote features 16–18 h after fertilization. Female pronucleus originates near the second polar body, whereas male pronucleus appears at the center of the cytoplasm. Following their formation, the female pronucleus migrates towards the male one until they are in close apposition. Nucleolar precursor bodies (NPB), randomly allocated within the pronuclei, appear shortly after fertilization and persist throughout the first cell cycles [6]. Unlike what was initially stated [7], the NPB are not precursors of nucleoli and they structurally support the formation of functional nucleoli when transcription starts in early embryos [6]. In the nucleoli, pre-ribosomal RNA (rRNA) synthesis occurs, the newly synthesized rRNAs are necessary for the translational process when the embryonic genome fully activates [8]. The progressive  polarization of NPB controls the design of the embryonic axis, a fundamental step for cell determination in the developing embryo [9]. Alterations of these strictly related events may have abnormal consequences, including fertilization failure and uneven cleavage.

Starting from the first observations on PN, different classification systems have been proposed taking into account the PN size, the NPB position and alignment [10]. Although PN scores have been correlated with embryo development, pregnancy and implantation, to date there are conflicting evidences on the relationship between zygote morphology and IVF outcomes [10]. One of the main limitations of current literature is the use of different zygote grading systems. To this regard, in 2011 the ESHRE-Alpha consensus defined three PN categories to standardize the zygote assessment: symmetrical, non-symmetrical and abnormal [1]. The symmetrical category includes zygotes showing two polar bodies, two centrally located and juxtaposed pronuclei, equal size and equivalent numbers and size of NPB equatorially aligned at the membrane juxtaposition. All the zygotes that do not have this ideal configuration belong to the non-symmetrical category. The abnormal category includes zygotes with no or one NPB. No studies verified the efficacy of such classification so far.

The aim of this retrospective study was to assess whether the consensus ESHRE system for the PN scoring could be a useful additional criterion to improve prediction of the embryo implantation potential.

## Methods

### Study design, size, duration

This is a retrospective, longitudinal, observational, cohort study. We included 3004 zygotes from 555 women (mean age: 35.6 years; range: 21–43) enrolled at our center between January 2014 and June 2019. The zygotes to be included in the study were selected on the basis of knowledge of their outcome: when 2 embryos from zygotes with different PN scores were transferred and only one implanted, these zygotes were excluded from the study. We included only homologous cycles using fresh eggs and ejaculated sperm. Another exclusion criterion was standard IVF, in order to standardize the fertilization check timing.

Embryo transfers (ET) were routinely performed on day 2–3 and, when available, two cleavage-stage embryos were transferred. Surplus embryos were cultured up to day 5–7 and those that developed up to blastocyst stage were cryopreserved. The ET were performed on day 5 in those cases with at least four good quality cleavage-stage embryos owing to the benefit from further observation in selecting the best embryos to transfer. On day 5 only elective single-embryo transfer were performed. In the study we included: (i) cleavage-stage ET; (ii) blastocyst-stage ET. Implantation success of each transferred embryo was defined as fetal cardiac activities at 12 weeks of gestation; miscarriages were excluded.

A STROBE (STrengthening the Reporting of OBservational studies in Epidemiology) checklist guideline is reported in Additional File 1.

### Outcomes measures

The primary outcome was implantation success in relation to the PN score of (i) cleavage-stage embryos; (ii) blastocyst-stage embryos, and their predictive factors. The secondary outcomes were cleavage rate, quality of embryos, blastocyst development in relation to the PN score, and their predictive factors. We also evaluated the outcome of the newborns collecting their birthweights (expressed as percentile and SD-score for gestational age, according the World Health Organization reference curves).

### Patients treatment

Standard controlled ovarian stimulation protocols were used. Pituitary suppression was achieved with either Gonadotropin Releasing Hormone agonists or antagonists. Stimulation with gonadotropins was monitored by measuring serum estradiol levels and follicle growth. The trigger was either recombinant or urinary human chorionic gonadotropin or agonist trigger. Cumulus-oocyte complexes were collected 36 h later, washed in Sydney IVF Gamete buffer (Cook Medical, Sydney, Australia) and immediately incubated in Sydney IVF Fertilization medium (Cook Medical) at 37 °C in a humidified atmosphere of 6 % CO_2_, 5 % O_2_ (Galaxy 48R incubators, New Brunswick Scientific, Edison, NJ, USA).

### Standard embryo culture

 After 2 h of incubation, the oocytes were denuded in HEPES-buffered medium (Sydney IVF Gamete medium, Cook Medical) containing 20 IU/ml of Hyaluronidase (Origio, Målov, Denmark). ICSI was performed immediately after denudation. Sperm samples were treated with a two-layer density gradient system (Sydney IVF Sperm Gradient, Cook Medical) or via Swim-up using Sydney IVF Gamete Buffer (Cook Medical). Incubations were performed at 37 °C in a humidified atmosphere of 6 % CO_2_, 5 % O_2_ (Galaxy 48R incubator; New Brunswick Scientific). Fertilization was assessed 16–18 h after injection (PN score 1: symmetrical, score 2: non-symmetrical, score 3: abnormal) [1], and embryos with two pronuclei were individually cultured from day 1 to day 3 into Sydney IVF Cleavage medium (Cook Medical) and from day 3 to day 5–7 in Sydney IVF Blastocyst medium (Cook Medical).

Standard day 2–3 embryo and blastocyst morphological assessment was carried out according to the current consensus system [1]. Arrested embryos were non-viable embryos in which development arrested for at least 24 h, or in which all the cells degenerated or fragmented.

### Statistical analyses

Descriptive statistics are reported as means ± standard deviation (SD) for continuous variables and as absolute frequencies and percentages for categorical variables. Comparisons of age, cleavage rate, quality of embryos, blastocyst development, implantation success among PN score groups were evaluated by chi-square test or Kruskal-Wallis test as appropriate.

Potential predictors (PN score, cleavage-stage embryo morphology, patient’s age, day of ET) of the study primary outcome (implantation *versus* non implantation) were first tested in univariable analysis using generalized estimating equations (GEE) with a logit link function for estimating the unadjusted odds ratio (OR) and 95 % confidence interval (95 % CI) for the success of the implantation. Then, variables potentially associated with the success of the implantation in univariable comparisons (*P* < 0.05) were included in a multivariable analysis for calculating the adjusted OR for the success of the implantation. GEE models were used in order to take into account the correlation between embryos originated from the same patient and an unstructured correlation matrix was used as correlation structure.

Sensitivity analyses excluding PN score 3 embryos (*n* = 21) or including only the top quality embryo (*n* = 860) were performed.

Analyses were carried out by MedCalc® software (Mariakerke, Belgium) and SAS 9.4 (SAS Institute, Cary, NC, USA). A *P* value < 0.05 was considered significant.

## Results

Among the embryos originated from the 3004 zygotes enrolled in the study, 1163 embryos were transferred into uterus at their cleavage-stage, 1490 were cultured up to day 5–7, 232 arrested on day 3, 9 were cryopreserved on day 2–3; 110 zygotes did not cleave. Among the 1490 embryos whose culture was extended up to day 5–7, 516 became blastocysts: 123 were transferred into uterus on day 5 and 393 were cryopreserved (226 on day 5, 156 on day 6, and 11 on day 7).

### Relationship between PN score and embryo quality at cleavage stage

**Fig. 1:**
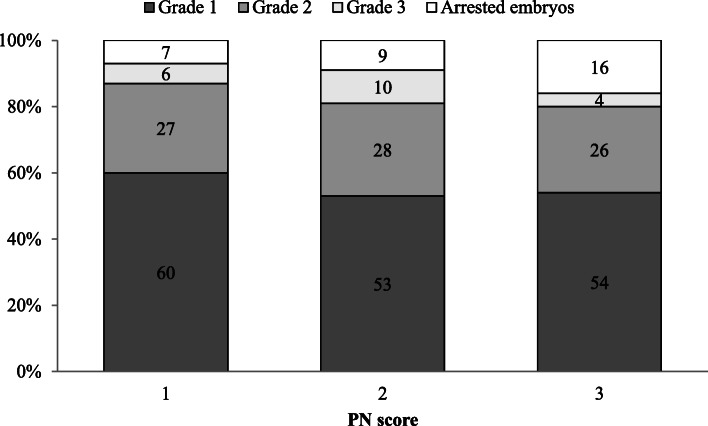
Stacked bar chart showing the morphological grade of cleavage-stage embryos based on their PN score

A total of 2280 (76 %) score 1, 645 (21 %) score 2, and 79 (3 %) score 3 zygotes were obtained. There was not a significant difference in patients’ age, cleavage rate and day 2–3 embryo morphology among the three PN score groups (Table 1). Moreover, the PN score did not always correlate with the embryo grade: only 60 % top quality zygotes formed grade 1 embryos, and 54 % poor quality zygotes (score 3) became high quality embryos (Fig. 1).

**Table 1:** Relationship between PN score and embryo quality parameters

PN score	1	2	3
**N. zygotes (%)**	2280 (76 %)	645 (21 %)	79 (3 %)
**Patients’ age (mean** **±** **SD, years)**	35.7 ± 4.4	35.3 ± 4.8	34.4 ± 4.1
**Cleavage rate**	97 %	96 %	94 %
**N. grade 1 embryos (%)**	1325/2280 (58 %)	320/645 (52 %)	42/79 (53 %)
**N. grade 2 embryos (%)**	578/2280 (25 %)	173/645 (27 %)	18/79 (23 %)
**N. grade 3 embryos (%)**	139/2280 (6 %)	70/645 (11 %)	3/79 (4 %)
**N. arrested cleavage-stage embryos (%)**	161/2280 (6 %)	55/645 (8 %)	11/79 (14 %)
**Blastulation rate**	384/1106 (35 %)	114/346 (33 %)	18/38 (47 %)

### Relationship between PN score and blastocyst development

Blastocyst development rate was similar among the three PN score-derived embryo groups, without any statistically significant differences (Table 1). A total of 1106 PN score 1-, 346 score 2-, and 38 score 3-derived embryos were placed in extended culture. We did not find any reduction of blastulation rates in score 2–3 (132/384, 34 %) groups respect to score 1 (384/1106, 35 %) (Table 1). Notably, a single NPB was observed in the majority of the abnormal score 3 PN category (75/79 cases) and 45 % of cases (17/38) developed up to blastocyst stage when prolonged culture was performed.

**Fig. 2:**
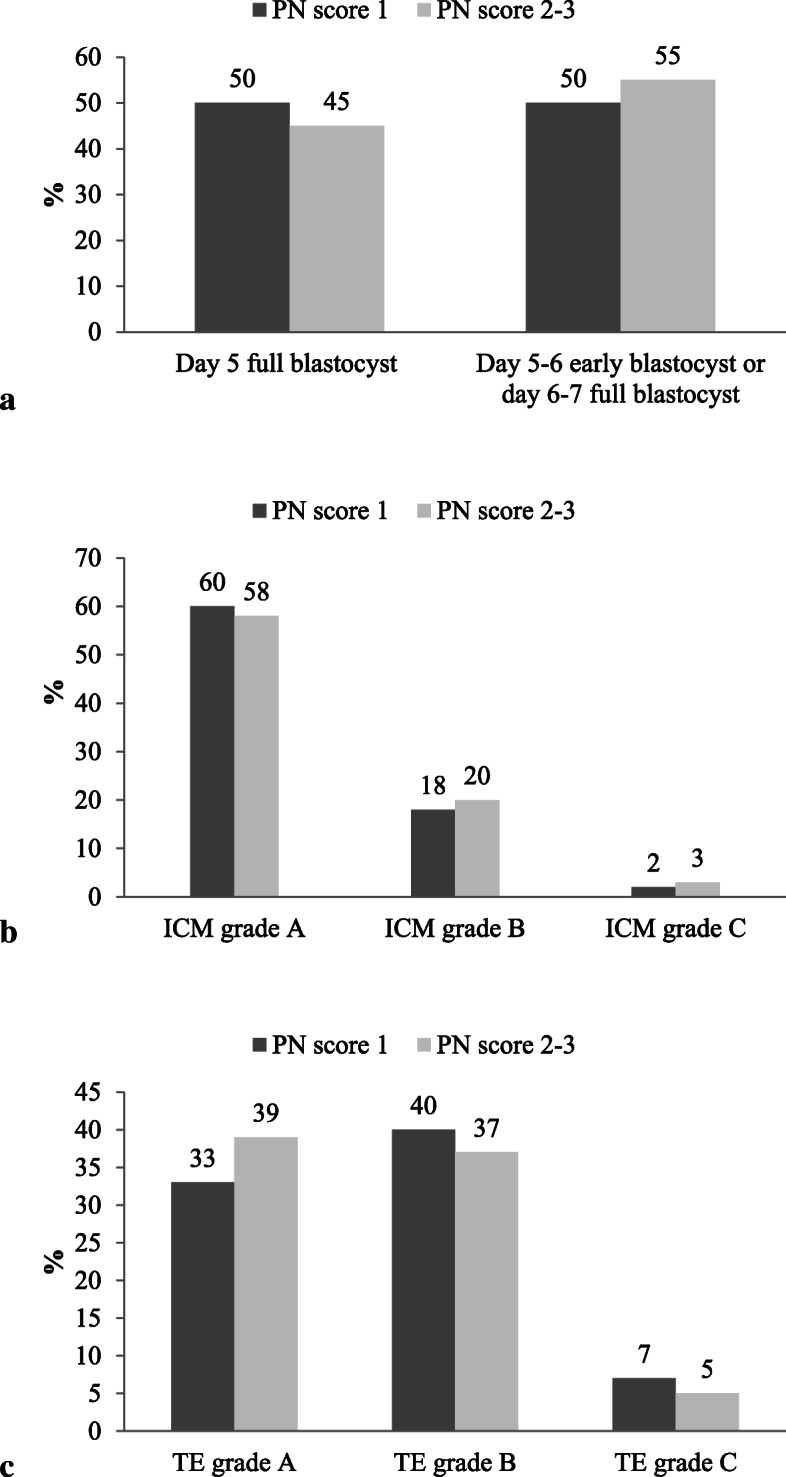
Bar graphs comparing blastocysts parameters based on their PN score. Legend: Panel **a**: timing of blastulation; Panel **b**: ICM morphological grade; Panel **c**: TE morphological grade

First, we compared PN score and timing of blastulation. Among the 384 PN score 1-derived embryos that reached the blastocyst stage, 191 (50 %) showed blastocoele expansion (grade ≥ 3) on day 5; 77 (20 %) embryos were early blastocysts on day 5, and 116 (30 %) full blastocysts later on day 6. Similarly, among the 132 PN score 2- and 3-derived embryos that reached the blastocyst stage, 59 (45 %) showed blastocoele expansion on day 5, 25 (19 %) were early blastocysts on day 5 (*n* = 22) or day 6 (*n* = 3), and 48 (36 %) full blastocysts later on day 6 (Fig. 2a).

Second, we stratified embryos according to ICM and TE morphological grade. From PN score 1-derived embryos there were 232/384 (60 %) ICM grade A blastocysts (153 on day 5, 79 on day 6–7), 68/384 (18 %) ICM grade B blastocysts (35 on day 5, 33 on day 6–7), and 7/384 (2 %) ICM grade C blastocysts (3 on day 5, 4 on day 6). Similarly, from PN score 2 and 3-derived embryos there were 77/132 (58 %) ICM grade A blastocysts (42 on day 5, 35 on day 6–7), 26/132 (20 %) ICM grade B blastocysts (15 on day 5, 11 on day 6–7), and 4/132 (3 %) ICM grade C blastocysts (2 on day 5 and 2 on day 6–7) (Fig. 2b).

From PN score 1-derived embryos there were 127/384 (33 %) TE grade A blastocysts (89 on day 5, 38 on day 6–7), 154/384 (40 %) TE grade B blastocysts (94 on day 5, 60 on day 6–7), and 26/384 (7 %) TE grade C blastocysts (8 on day 5, 18 on day 6–7). From PN score 2- and 3-derived embryos there were 51/132 (39 %) TE grade A blastocysts (32 on day 5, 19 on day 6), 49/132 (37 %) TE grade B blastocysts (24 on day 5, 25 on day 6–7), and 7/132 (5 %) TE grade C blastocysts (3 on day 5, 4 on day 6–7) (Fig. 2c).

### Association between PN score and implantation success

We analyzed 1150 transferred embryos from 476 patients, in which the outcome for all embryos was known. Due to the paucity of score 3 PN zygotes, we merged score 2 (*n* = 197) and score 3 (*n* = 21) PN-derived embryos in the subsequent analyses.

**Fig. 3:**
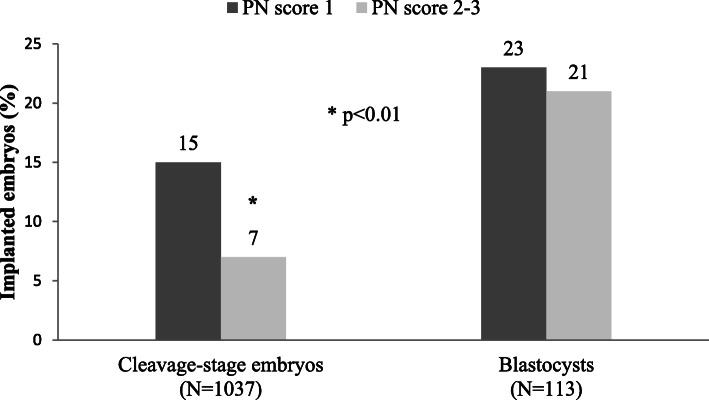
Bar graph comparing the percentage of embryo implanted accordingly to their PN score

A greater implantation success was observed for PN score 1- respect to PN score 2-3-derived embryos (15 and 9 %, respectively, *P* = 0.0121). This behavior was observed for both ET types, with statistically significant differences reached for the cleavage-stage group (15 % vs. 7 %, *P* = 0.0043), due to its numerousness (Fig. 3). Three out of 21 transferred abnormal score 3 PN zygotes - specifically with a single NPB - were observed in the implanted embryo group.

Factors associated to the implantation at univariable and multivariable analysis are reported in Table 2. A PN score 1 (OR = 1.76; 95 % CI:1.31–2.37, *P* = 0.0002), a cleavage stage embryo morphology equal to 1 (OR = 4.78; 95 % CI:1.84–12.42, *P* = 0.001), age less than 35 years (OR = 2.85; 95 % CI: 1.91–4.24, *P* < 0.0001) and ET on day 5 (OR = 2.12; 95 % CI: 1.32–3.39, *P* = 0.002) were factors associated to a successful implantation. A cleavage stage morphology equal to 2 showed a trend for the success of the implantation (OR = 2.43; 95 % CI: 0.90–6.55, *P* = 0.08). The multivariable analysis confirmed the findings of the unadjusted associations.

**Table 2:** Univariable and multivariable analysis of predictors of implantation success for all embryos (*n* = 1150)

		Unadjusted OR (95 % CI)	*p*-value	Adjusted OR (95 % CI)	*p*-value
PN score	2–3 (ref)	-			
	1	1.76 (1.31–2.37)	0.0002	1.83 (1.34–2.50)	0.0001
Cleavage stage morphology	3 (ref)	-			
	1	4.78 (1.84–12.42)	0.001	4.20 (1.68–10.48)	0.002
	2	2.43 (0.90–6.55)	0.080	2.16 (0.84–5.59)	0.111
Age	≥ 35 (ref)	-			
	< 35	2.85 (1.91–4.24)	< 0.0001	2.87 (1.93–4.27)	< 0.0001
ET day	Day 2–3 (ref)	-			
	Day 5	2.12 (1.32–3.39)	0.002	1.79 (1.09–2.93)	0.020

A sensitivity analysis excluding PN score 3 embryos (*n* = 21) was also performed and no differences in unadjusted and adjusted ORs were found (see Additional file 2).

In a sensitivity analysis including only the top quality embryos (n = 860) we did not find any differences in unadjusted and adjusted ORs (Table 3).

**Table 3:** Univariable and multivariable analysis of predictors of implantation success for top quality embryos (*n* = 860)

		Unadjusted OR (95 % CI)	*p*-value	Adjusted OR (95 % CI)	*p*-value
PN score	2–3 (ref)	-			
	1	1.60 (1.12–2.30)	0.011	1.68 (1.17–2.42)	0.005
Age	≥ 35 (ref)	-			
	< 35	2.81 (1.86–4.25)	< 0.0001	2.80 (1.85–4.23)	< 0.0001
ET day	Day 2–3 (ref)	-			
	Day 5	1.88 (1.16–3.04)	0.010	1.71 (1.03–2.84)	0.039

### Perinatal characteristics of newborns

We excluded from this analysis the outcome of ET of two embryos with discordant PN scores that resulted in delivery of only one baby. A total of 104 neonatal outcomes from PN score 1 embryos (of which 15 from twin gestations) and 4 from PN score 2 embryos (all from single pregnancies) were available. As detailed in Additional file 3 no statistical analyses could be performed due to the small number of evaluable newborns in PN score 2 group. We observed absence of twin pregnancies in PN score 2 category: this accounts for the higher birthweights and longer gestational period in PN score 2 respect to PN score1 group. In PN score 1 group, 8 pregnancies started as dichorionic-diamniotic twin pregnancies, but resulted in the live birth of only one of the fetuses. In fact, in 7 cases there was a spontaneous first trimester abortion of one twin, and in one case the patient opted for an elective pregnancy termination for a congenital mega bladder. Among singleton pregnancies, there was another elective termination for a major central nervous system malformation. No stillbirths as well as no malformations were recorded among the newborns of both score groups.

## Discussion

To date, literature data about the correlation among zygote morphology, biological and clinical outcomes are inconclusive, mainly due to different methods used for PN scoring, time of PN observation, and insemination procedure [10].

This is the first study that evaluated the prognostic effect of the ESHRE consensus PN scoring system by a multivariable analysis. We did not find any relationship among PN score, embryo quality at cleavage and blastocyst stages, and blastulation rate, in line with some previous studies [11–17]. Intriguingly, half embryos from a single NPB zygote developed up to blastocyst stage and three successfully implanted. These data suggest that embryos derived from abnormal PN zygotes have some development potential, as described in literature using time-lapse imaging [18].

We demonstrated that the PN score improves prediction of implantation of cleavage-stage good morphology embryos. Moreover, PN scores 2 and 3 were associated with a lower implantation success, even though the morphology of the embryos was good. Based on these findings we argue that PN score may provide a non-invasive, early criterion helpful for selecting the best embryo(s) for transfer, particularly when multiple embryos of similar quality are available.

Overall, the multivariable analysis including potential confounding factors associated with the occurrence of pregnancy found that the PN score 1- along with top quality cleavage stage embryo morphology, patient’s age less than 35 years and ET on day 5 - was predictive of embryos’ implantation success.

The 2 and 3 PN patterns may be characterized by asynchrony in the formation and polarization of pronuclei. Such alteration of sequential, linked events can be at the origin of chromosomal abnormalities, whose consequences may appear at the implantation phase, after the embryonic genome activation [19–21]. This proposition would explain why we found a significant positive association between PN score 1 and implantation, without any evident effect of PN morphology on *in vitro* embryo developmental potential.

At pronuclear stage check, most embryos are at S or G2 phase when chromosomes are interconnected via nucleoli. As it has been already reported by some authors [22–24], if zygotes have different PN size and non-synchronous NPB there is an increased risk of embryo aneuploidy, due to aberrant chromosome duplication and division. Therefore, in absence of a genetic embryo assessment the PN score may help selection of those top quality embryos that have a chromosomal euploid set.

As observed in this study, top quality zygotes can become low quality embryos as well as top quality embryos can develop from low quality zygotes. In other words, neither zygote, nor embryo morphology alone are fully predictive of IVF outcome. Therefore, we believe that a combination of assessments and scores, including the PN score, may be helpful in non-invasive embryo selection.

Time-lapse monitoring of embryo development showed that PN morphology changes within a short time, at 16–20 h after ICSI, mostly from an asymmetry of NPB towards a symmetric or perfectly aligned distribution [18]. Therefore, a single microscopy observation may be misleading and such changes may partially explain the contradictory literature, where some authors reported no benefit when static observations of PN were performed [14, 25, 26]. Despite the dynamicity of the PN formation, our findings could be nonetheless useful for the majority of laboratories without of time-lapse technology availability. Certainly, the lack of standardization in the observation timing remains a critical issue for PN scoring, including the variability due to the method used for insemination. In fact, pronucleus development has an average delay of 4 h after conventional IVF as compared to ICSI, likely because the spermatozoon needs time to pass through the cumulus and corona cells and the zona pellucida [27]. This is the reason why we have excluded standard IVF cycles. We believe that the retrospective nature of this study could hardly have affected the reliability of our findings since at our laboratory all procedures were done by the same embryologists, who did not change throughout the duration of the study and applied the same protocols, including consistent timing of PN assessment.

## Conclusions

This is the first study of correlation between PN morphology and embryo implantation success, applying the PN score system proposed by ESHRE and a multivariable analysis which evaluated various potential confounding factors. Although validation through randomized perspective studies is needed, our findings suggest that the PN score could represent the earliest point at which the quality of the fertilized oocyte can be non-invasively evaluated and that the PN score may be routinely included among criteria for embryo evaluation. In this way, an evaluation based on the combination of both zygote and cleavage-stage morphology could assist in selecting the top quality embryo(s) with the highest chances of implantation. This could be of great value for all laboratories performing clinical IVF without any pre-implantation genetic testing means.

## Supplementary Information

**Additional file 1.** STROBE reporting guideline of the study.

**Additional file 2.** Multivariable logistic analysis: predictors of implantationof PN score 1- vs. PN score 2-embryos (*n*=1084).

**Additional file 3.** Neonatal characteristics of newborns.

## Data Availability

The datasets analyzed during the current study are available from the corresponding author upon request.

## Abbreviations

HEPES: 4-(2-hydroxyethyl)-1-piperazineethanesulfonic acid
ET: Embryo transfers
ESHRE: European Society of Human Reproduction and Embryology
ICM: Inner cell mass
ICSI: Intra cytoplasmic sperm injection
IVF: In vitro fertilization
NPB: Nucleolar precursor bodies
PN: Pronuclear
rRNA: Ribosomal RNA
SD: Standard deviation
TE: Trophectoderm
