# Supplementary material for: Pronuclear score improves prediction of embryo implantation success in ICSI cycles
Source: BMC Pregnancy Childbirth. 2021 May 5;21:361. doi: 10.1186/s12884-021-03820-7 (PMC8097973; doi:10.1186/s12884-021-03820-7)
Supplement: Supplementary file 1 — Additional file 1. STROBE reporting guideline of the study. [file 12884_2021_3820_MOESM1_ESM.docx]

**Additional File 1.** STROBE reporting guideline of the study.

|  | Item No. | Recommendation | Page  No. | Relevant text from manuscript |
| --- | --- | --- | --- | --- |
| **Title and abstract** | 1 | (*a*) Indicate the study’s design with a commonly used term in the title or the abstract | 2 | Line 10 |
|  |  | (*b*) Provide in the abstract an informative and balanced summary of what was done and what was found | 2  3 | Lines 10-25  Lines 1-2 |
| Introduction | | | |  |
| Background/rationale | 2 | Explain the scientific background and rationale for the investigation being reported | 4  5 | Lines 2-26  Lines 1-10 |
| Objectives | 3 | State specific objectives, including any prespecified hypotheses | 5 | Lines 11-13 |
| Methods | | | |  |
| Study design | 4 | Present key elements of study design early in the paper | 5 | Line 16 |
| Setting | 5 | Describe the setting, locations, and relevant dates, including periods of recruitment, exposure, follow-up, and data collection | 5 | Lines 17-18 |
| Participants | 6 | (*a*) *Cohort study*—Give the eligibility criteria, and the sources and methods of selection of participants. Describe methods of follow-up  *Case-control study*—Give the eligibility criteria, and the sources and methods of case ascertainment and control selection. Give the rationale for the choice of cases and controls  *Cross-sectional study*—Give the eligibility criteria, and the sources and methods of selection of participants | 5 | Lines 18-22 |
|  |  | (*b*) *Cohort study*—For matched studies, give matching criteria and number of exposed and unexposed  *Case-control study*—For matched studies, give matching criteria and the number of controls per case |  |  |
| Variables | 7 | Clearly define all outcomes, exposures, predictors, potential confounders, and effect modifiers. Give diagnostic criteria, if applicable | 6  7 | Lines 7-12  Lines 13-14 |
| Data sources/ measurement | 8* | For each variable of interest, give sources of data and details of methods of assessment (measurement). Describe comparability of assessment methods if there is more than one group | 7 | Lines 1-9 |
| Bias | 9 | Describe any efforts to address potential sources of bias | 7 | Lines 13-21 |
| Study size | 10 | Explain how the study size was arrived at | 5 | Lines 16-18 |

| Quantitative variables | 11 | Explain how quantitative variables were handled in the analyses. If applicable, describe which groupings were chosen and why | 7 | Lines 10-12 | |
| --- | --- | --- | --- | --- | --- |
| Statistical methods | 12 | (*a*) Describe all statistical methods, including those used to control for confounding | 7 | Lines 8-23 | |
|  |  | (*b*) Describe any methods used to examine subgroups and interactions | 7 | Lines 13-21 | |
|  |  | (*c*) Explain how missing data were addressed |  |  | |
|  |  | (*d*) *Cohort study*—If applicable, explain how loss to follow-up was addressed  *Case-control study*—If applicable, explain how matching of cases and controls was addressed  *Cross-sectional study*—If applicable, describe analytical methods taking account of sampling strategy |  |  | |
|  |  | (*e*) Describe any sensitivity analyses | 7 | Lines 22-23 | |
| Results | | | | | |
| Participants | 13* | (a) Report numbers of individuals at each stage of study—eg numbers potentially eligible, examined for eligibility, confirmed eligible, included in the study, completing follow-up, and analysed | 8 | Lines 2-6 | |
|  |  | (b) Give reasons for non-participation at each stage | 9  10 | Lines 14-15  Lines 1-12 | |
|  |  | (c) Consider use of a flow diagram |  |  | |
| Descriptive data | 14* | (a) Give characteristics of study participants (eg demographic, clinical, social) and information on exposures and potential confounders | Table 1 | | |
|  |  | (b) Indicate number of participants with missing data for each variable of interest |  | |  |
|  |  | (c) *Cohort study*—Summarise follow-up time (eg, average and total amount) | 5  6  7 | | Lines 23-26  Lines 1-4  Lines 1-7 |
| Outcome data | 15* | *Cohort study*—Report numbers of outcome events or summary measures over time | 8  9  10 | | Lines 7-8, 13-17, 20-25  Lines 1-22  Lines 12-23 |
|  |  | *Case-control study—*Report numbers in each exposure category, or summary measures of exposure |  | |  |
|  |  | *Cross-sectional study—*Report numbers of outcome events or summary measures |  | |  |
| Main results | 16 | (*a*) Give unadjusted estimates and, if applicable, confounder-adjusted estimates and their precision (eg, 95% confidence interval). Make clear which confounders were adjusted for and why they were included | 9  10  Table 2  Table 3  Additional File 2  Additional File 3 | | Lines 23-26  Lines 1-10 |
|  |  | (*b*) Report category boundaries when continuous variables were categorized | Tables 2-3 (for age variable)  Additional File 2 (for age variable) | | |
|  |  | (*c*) If relevant, consider translating estimates of relative risk into absolute risk for a meaningful time period |  | |  |

| Other analyses | 17 | Report other analyses done—eg analyses of subgroups and interactions, and sensitivity analyses | 10  Table 3  Additional File 2 | | Lines 7-10 |
| --- | --- | --- | --- | --- | --- |
| Discussion | | | | | |
| Key results | 18 | Summarise key results with reference to study objectives | 11 | Lines 5-7, 10-15 | |
| Limitations | 19 | Discuss limitations of the study, taking into account sources of potential bias or imprecision. Discuss both direction and magnitude of any potential bias | 12 | Lines 7-20 | |
| Interpretation | 20 | Give a cautious overall interpretation of results considering objectives, limitations, multiplicity of analyses, results from similar studies, and other relevant evidence | 12 | Lines 7-20 | |
| Generalisability | 21 | Discuss the generalisability (external validity) of the study results | 12  13 | Lines 25-26  Lines 1-5 | |
| Other information | |  | | | |
| Funding | 22 | Give the source of funding and the role of the funders for the present study and, if applicable, for the original study on which the present article is based | 14 | Lines 5-6 | |

*Give information separately for cases and controls in case-control studies and, if applicable, for exposed and unexposed groups in cohort and cross-sectional studies.
