# Supplementary material for: Pronuclear score improves prediction of embryo implantation success in ICSI cycles
Source: BMC Pregnancy Childbirth. 2021 May 5;21:361. doi: 10.1186/s12884-021-03820-7 (PMC8097973; doi:10.1186/s12884-021-03820-7)
Supplement: Supplementary file 2 — Additional file 2. Multivariable logistic analysis: predictors of implantationof PN score 1- vs. PN score 2-embryos (n=1084). [file 12884_2021_3820_MOESM2_ESM.docx]

**Additional file 2**. Multivariable logistic analysis: predictors of implantation of PN score 1- vs. PN score 2-embryos (n=1084).

|  |  | **Unadjusted OR (95% CI)** | **p-value** | **Adjusted OR (95% CI)** | **p-value** |
| --- | --- | --- | --- | --- | --- |
| PN score | 2 (ref) | - |  |  |  |
|  | 1 | 1.73 (1.26-2.37) | 0.0007 | 1.76 (1.26-2.45) | 0.0008 |
| Cleavage stage morphology | 3 (ref) | - |  |  |  |
|  | 1 | 4.96 (1.88-13.12) | 0.001 | 4.35 (1.72-11.04) | 0.002 |
|  | 2 | 2.54 (0.93-6.98) | 0.070 | 2.30 (0.87-6.03) | 0.092 |
| Age | ≥35 (ref) | - |  |  |  |
|  | <35 | 2.87 (1.93-4.27) | <0.0001 | 2.85 (1.92-4.24) | <0.0001 |
| ET day | Day 2-3 (ref) | - |  |  |  |
|  | Day 5 | 2.16 (1.35-3.46) | 0.001 | 1.82 (1.12-2.98) | 0.016 |
