# Supplementary material for: Pronuclear score improves prediction of embryo implantation success in ICSI cycles
Source: BMC Pregnancy Childbirth. 2021 May 5;21:361. doi: 10.1186/s12884-021-03820-7 (PMC8097973; doi:10.1186/s12884-021-03820-7)
Supplement: Supplementary file 3 — Additional file 3. Neonatal characteristics of newborns. [file 12884_2021_3820_MOESM3_ESM.docx]

|  | **PN 1** | **PN 2** |
| --- | --- | --- |
| N. live births | 104 | 4 |
| N. lost follow-up | 6 | 1 |
| N. ongoing pregnancies | 0 | 0 |
| Birthweight (grams) |  |  |
| Total | 2902.8 + 598.9 | 3443.8 + 328.5 |
| Singletons | 3130.8 + 516.8 | 3443.8 + 328.5 |
| Twins | 2340.4 + 376.4 | - |
| N. birthweight <2,500 g |  |  |
| Total | 24 | 0 |
| Singletons | 7 | 0 |
| Twins | 17 | - |
| Gestational age (weeks) |  |  |
| Total | 37.9 + 2.3 | 40.3 + 0.5 |
| Singletons | 38.7 + 2.0 | 40.3 + 0.5 |
| Twins | 35.9 + 1.9 | - |
| N. prematurity <37 weeks |  |  |
| Total | 23 | 0 |
| Singletons | 9 | 0 |
| Twins | 14 | - |
| Birthweight centiles |  |  |
| Total | 43.5 + 30.1 | 52.5 + 27.3 |
| Singletons | 46.5 + 30.3 | 52.5 + 27.3 |
| Twins | 35.9 + 28.9 | - |
| SD-score |  |  |
| Total | -0.2 + 1.1 | 0.1 + 0.8 |
| Singletons | -0.1 + 1.0 | 0.1 + 0.8 |
| Twins | -0.5 + 1.1 | - |

**Additional file 3.** Neonatal characteristics of newborns

Values are mean ± SD unless otherwise stated
